# Bladder cancer prognosis using deep neural networks and histopathology images

**DOI:** 10.1016/j.jpi.2022.100135

**Published:** 2022-08-28

**Authors:** Wayner Barrios, Behnaz Abdollahi, Manu Goyal, Qingyuan Song, Matthew Suriawinata, Ryland Richards, Bing Ren, Alan Schned, John Seigne, Margaret Karagas, Saeed Hassanpour

**Affiliations:** a Department of Computer Science, Dartmouth College, Hanover, NH, USA; b Department of Biomedical Data Science, Dartmouth College, Hanover, NH, USA; c Department of Pathology and Laboratory Medicine, Dartmouth-Hitchcock Medical Center, Lebanon, NH, USA; d Department of Surgery, Division of Urology, Dartmouth-Hitchcock Medical Center, Lebanon, NH, USA; e Department of Epidemiology, Dartmouth College, Hanover, NH, USA

**Keywords:** Bladder cancer, Computational pathology, Convolutional neural networks

## Abstract

**Background:**

Recent studies indicate that bladder cancer is among the top 10 most common cancers in the world (Saginala et al. 2022). Bladder cancer frequently reoccurs, and prognostic judgments may vary among clinicians. As a favorable prognosis may help to inform less aggressive treatment plans, classification of histopathology slides is essential for the accurate prognosis and effective treatment of bladder cancer patients. Developing automated and accurate histopathology image analysis methods can help pathologists determine the prognosis of patients with bladder cancer.

**Materials and methods:**

In this study, we introduced Bladder4Net, a deep learning pipeline, to classify whole-slide histopathology images of bladder cancer into two classes: low-risk (combination of PUNLMP and low-grade tumors) and high-risk (combination of high-grade and invasive tumors). This pipeline consists of four convolutional neural network (CNN)-based classifiers to address the difficulties of identifying PUNLMP and invasive classes. We evaluated our pipeline on 182 independent whole-slide images from the New Hampshire Bladder Cancer Study (NHBCS) (Karagas et al., 1998; Sverrisson et al., 2014; Sverrisson et al., 2014) collected from 1994 to 2004 and 378 external digitized slides from The Cancer Genome Atlas (TCGA) database (https://www.cancer.gov/tcga).

**Results:**

The weighted average F1-score of our approach was 0.91 (95% confidence interval (CI): 0.86–0.94) on the NHBCS dataset and 0.99 (95% CI: 0.97–1.00) on the TCGA dataset. Additionally, we computed Kaplan–Meier survival curves for patients who were predicted as high risk versus those predicted as low risk. For the NHBCS test set, patients predicted as high risk had worse overall survival than those predicted as low risk, with a log-rank p-value of 0.004.

**Conclusions:**

If validated through prospective trials, our model could be used in clinical settings to improve patient care.

## Background

Recent studies indicate that bladder cancer is among the top 10 most common cancers in the world.^1^ Urothelial carcinoma accounts for most cases of bladder cancer. Approximately, 75–85% of patients with bladder cancer are classified as having nonmuscle invasive bladder cancer (NMIBC). Furthermore, approximately 50% of NMIBC patients experience more disease recurrences, and the treatment procedure is different from that of patients diagnosed with muscle-invasive bladder cancer (MIBC).^6^ Urothelial carcinomas are graded according to the degree of tumor cellular and architectural atypia. The cancer grade has an important role in deciding the treatment plan, so if not determined accurately, the patient may undergo unnecessary treatments. The World Health Organization (WHO) 1973 and World Health Organization/International Society of Urological Pathology (WHO/ISUP) classifications are widely used for tumor grading, but these methods have relatively high intra- and interobserver variabilities.^7^^,^^8^ Several studies compared different grading systems and their effect on choosing the best treatment.^9^, ^10^, ^11^, ^12^ A study used the WHO 1973 classification for evaluations and the interobserver agreement among 11 pathologists was slight to moderate (κ = 0.19 − 0.44).^13^ Another study measured interobserver agreement among six pathologists and showed that WHO/ISUP classification is slightly better than WHO 1973.^14^ Therefore, new methods should be sought to help pathologists diagnose bladder cancer.

The stage and grade of bladder tumors are important criteria in cancer treatment. The cancer stage consists of the location of the cancer cells and how far they have grown. Higher stages indicate whether the tumor has grown away from the surface. Urothelial carcinoma pathologic stages are named Ta (papillary tumor without invasion), TIS (carcinoma in situ (CIS)), T1 (tumor invades the connective tissue under the surface lining), T2 (tumor invades the muscle layer), T3 (tumor invades perivesical soft tissue), and T4 (extravesical tumor directly invades into other organs or structures). According to the WHO 2016 classification, NMIBC is divided into three groups: Ta, TIS, and T1, while MIBC is divided into T2, T3, and T4. In low-grade cancer cases, the cancer cells show morphology with less atypia, more closely resemble normal urothelial cells, and grow slowly. Clinical research has demonstrated that the most common bladder tumors are low-grade tumors.^15^ In contrast, high-grade cancer cells show more irregular and atypical morphology and can be found in both NMIBC and MIBC. It is essential to accurately differentiate between low- and high-grade cancers because different treatments are available for various grade tumors. For example, prompt treatment is required for high-grade cells in NMIBC to avoid the spread of cancer.

Papillary urothelial neoplasm of low malignant potential (PUNLMP) was first introduced by the WHO/ISUP in 1998 as a new entity of bladder cancer.^16^ PUNLMP and low-grade urothelial carcinoma are two bladder cancer types that are not easily distinguishable based on cell morphology under the microscope. Because of the similarities between these two cancer types, the pathologic diagnostic accuracy for their differentiation is approximately 50%.^17^ While the distinction between PUNLMP and low-grade urothelial carcinoma is deemed essential by some pathologists, recent studies have shown that separating PUNLMP and low-grade urothelial carcinoma is not clinically crucial.^9^ Importantly, high-grade tumor cells are found in various tumor stages, and MIBC is considered a type of high-grade cancer.

The histological classification of bladder cancer has significant implications for the prognosis and treatment of patients. Moreover, detecting and classifying histologic patterns such as PUNLMP and low-grade urothelial carcinoma under the microscope is a time-consuming and challenging task for pathologists. Manual classification of bladder cancer histological patterns has a high error rate due to the similarity of histological features. Therefore, clinical information such as the cancer stage is commonly used for a more accurate prognosis. Automated image analysis using deep learning techniques can assist pathologists in providing faster and more consistent results. Additionally, these techniques can be improved by providing new data and associated annotated labels by several pathologists so that the model can be trained based on the expert opinion of multiple pathologists.

Automated image analysis methods to classify and visualize various cancer patterns in high-resolution whole-slide images can help pathologists avoid errors and reduce their assessment time.^18^^,^^19^ In this study, we introduced a CNN-based model for the classification of urothelial bladder cancer based on whole-slide histopathology images to distinguish between low- and high-risk groups, where the low-risk class includes PUNLMP and low-grade cases, and the high-risk class includes high-grade and invasive cases.

## Materials and methods

### Datasets

For the model development and evaluation, we used images from the New Hampshire Bladder Cancer Study or NHBCS.^2^, ^3^, ^4^ Risk factors for bladder cancer have been widely explored in previous reports from this study.^20^ For external evaluation, we utilized histology images from The Cancer Genome Atlas (TCGA).^5^ The details of these datasets are included below.

### New Hampshire Bladder Cancer Study (NHBCS) dataset

This dataset contains 838 whole-slide images from 1994 to 2004 as part of the NHBCS.^21^ These hematoxylin and eosin (H&E)-stained surgical resection slides were digitized by Aperio AT2 scanners (Leica Biosystems, Wetzlar, Germany) at 20× magnification (0.50 μm/pixel).

We normalized the color intensity of patches and applied standard data augmentation methods, including random horizontal and vertical flips, random 90° rotations, and color jittering.

### The Cancer Genome Atlas (TCGA) dataset

We collected 378 whole-slide images from TCGA for external validation. The distribution of these whole-slide images used in this study is summarized in Table 1.

**Table 1:** Distribution of collected whole-slide images from four classes (PUNLMP, low-grade cases, high-grade cases, and IUC) and distribution of low-risk and high-risk images in our datasets.

Histologic subtype	Internal (NHBCS) training set	Internal (NHBCS) test set	External (TCGA) test set
Low-risk cases (PUNLMP + Low Grade)	248 (94 + 154)	107 (39 + 68)	11 (11 + 0)
High-risk cases (High Grade + IUC)	177 (144 + 33)	75 (62 + 13)	367 (0 + 367)
Total	425	182	378

### Data annotation

The tumor histologic subtypes in the NHBCS dataset were independently confirmed by two expert genitourinary pathologists from the Department of Pathology and Laboratory Medicine at Dartmouth–Hitchcock Medical Center (DHMC) based on a standard histopathology review. In the NHBCS dataset, 637 whole-slide images were categorized into papillary urothelial neoplasm of low malignant potential (PUNLMP), low-grade papillary urothelial carcinoma (low-grade, noninvasive), high-grade papillary urothelial carcinoma (high-grade, noninvasive), and invasive urothelial carcinoma (IUC). Among these slides, 34 were classified as carcinoma in situ (CIS). Because of the small number of available CIS cases, we removed them from our study. In addition, 31 cases were labeled as others, which were excluded from our study. We used 607 whole slides from four classes (PUNLMP, low-grade cases, high-grade cases, and IUC) in our analysis. We combined PUNLMP and low-grade whole-slide image cases into a single class because of their similarity, as they are both noninvasive and low-risk cancers. Additionally, high-grade and IUC cases were merged into one group because they are considered high-risk cancers.

We considered the grade heterogeneity during the annotation of the WSI of the urothelial lesions, especially the noninvasive low and high-grade papillary urothelial carcinoma. The criteria of WHO classification of low and high-grade papillary urothelial carcinoma^22^ were followed during our annotation, and the papillary urothelial carcinomas with ≥5% of high-grade features were classified as high-risk cases. We established the ground-truth labels for each whole-slide image in our NHBCS datasets based on the consensus opinion of the two pathologists. If there was any disagreement, an expert pathologist re-reviewed the whole-side image and resolved any disagreements. We randomly partitioned these slides into an internal training set of 425 slides (~70% of the NHBCS dataset) and an internal test set of 182 slides (~30% of the NHBCS dataset).

Two pathologists manually annotated the whole-slide images in our internal NHBCS training set using the Automated Slide Analysis Platform (ASAP).^23^ Regions of interest in each whole-slide image in our training set were annotated with bounding boxes at the highest resolution for each image. The annotated areas were split into smaller patches for training a patch-level classifier. As noted above, the ground-truth labels for whole-slide images in our internal NHBCS test set were based on the independent classification of two pathologists. The labels for the external TCGA test set were established based on the provided metadata from the TCGA database and additional confirmation by our study's expert pathologist.

### Bladder4Net: Deep learning pipeline

**Fig. 1:**
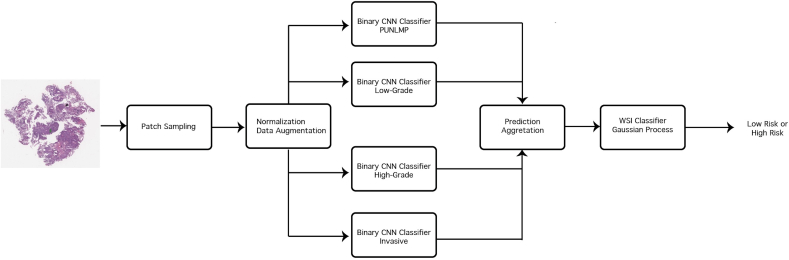
Overview of the Bladder4Net pipeline. Tissue patches were extracted from whole-slide images using the sliding-window method with 1/3 overlap after background, marker, and stain removal. Next, these patches were forward passed through four different binary CNN classifiers. The resulting predictions were grouped, and the ratio of patches from each class was computed. The above was used as input for a Gaussian process classifier to determine the final prediction: low risk or high risk.

In this study, we developed a deep learning-based model to distinguish between low- and high-risk bladder cancer cases, where the low-risk class includes PUNLMP and low-grade cases, and the high-risk class includes high-grade and invasive cases. This deep learning pipeline, named Bladder4Net, is shown in Fig. 1. We classified each patch in a whole-slide image with binary classifiers. The portion of patches classified as a subtype in a whole-slide image is included in a vector for all classes. Notably, the ratio of PUNLMP and low-grade patches is added to represent low-risk patches, and the ratio of high-grade and invasive patches is combined to represent high-risk patches. A Gaussian process classifier was trained on low- and high-risk patch ratios using the same training and test set partitioning used for training the CNN classifier. The details of this pipeline are included below.

### Patch classification

Analyzing large histology images using deep learning models requires substantial memory resources. Therefore, we split each whole-slide image into fixed-size patches (224 × 224 pixels) with 1/3 overlap. ResNet-18 is a light deep learning architecture. Therefore, it requires fewer computational resources to train, is faster during the inference time on large whole-slide images and is less prone to over-fitting. The Bladder4Net pipeline consists of four binary ResNet-18^24^ deep learning models that operate at the patch level for each class. We randomly select 10% of whole-slide images in the training partition for hyperparameter tuning to find the best hyperparameters during the training process. We selected patches in annotated areas for training and evaluating the patch-level classifiers. We normalized the color intensity of the patches and used standard data augmentation methods, including random vertical and horizontal flips and color jittering, whose parameters were selected based on the random subsampling of patches in each class. Our model was trained on 260 610 patches (an average of 613 patches per whole-image slide), including 107 379 high-risk and 153 231 low-risk patches. To address the class imbalance, we used a weighted random sampler method to generate the training batches. For model training, we trained a ResNet-18^24^ initialized using normal distribution initialization. All four models used the cross-entropy loss function and were trained for 100 epochs with an initial learning rate of 0.005 and decayed by a factor of 0.9 for each epoch.

### Whole-slide inference

**Fig. 2:**
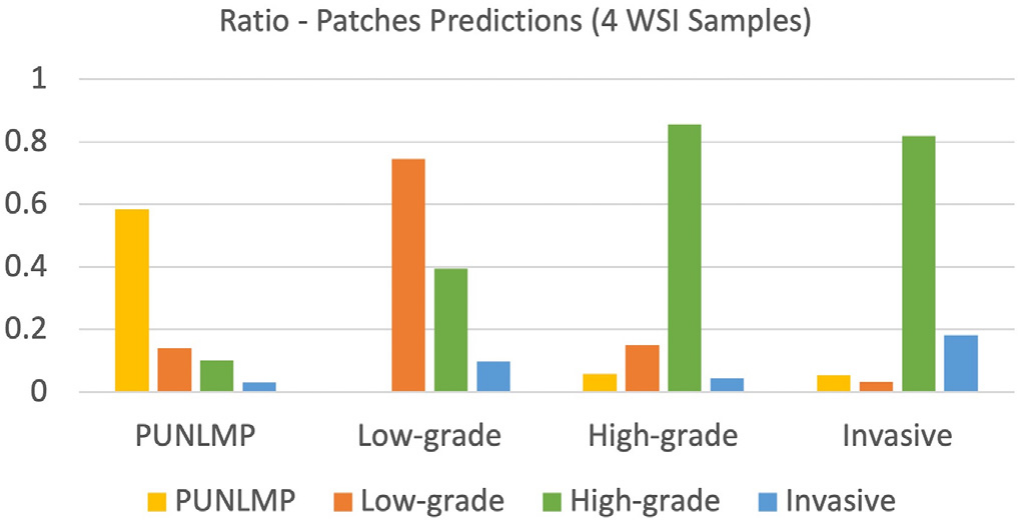
The ratio of patch predictions for four samples of whole-slide images (one per class). From left to right: the WSI corresponding to the PUNLMP class presents a high ratio of patch predictions for its respective class. The same behavior occurs for the low-grade class sample and high-grade class sample. However, the invasive sample does not follow this pattern because CNN classifiers cannot correctly predict this class; instead, they predict this class as a high-grade class.

To classify whole-slide images, we aggregated patch-level prediction outputs. For each whole-slide image, we preprocessed the image by removing the white background and color markers. To aggregate the patch-level predictions, the ratio of patches from each class to the total number of patches from a slide was computed per whole-slide image. Bladder cancer is progressive, and there are mixed types of cancer cells in many whole slides. Therefore, there were some low-risk patches in high-risk whole-slide images. We used a Gaussian process classifier for whole-slide inferencing. This classifier was trained on the patch ratios of whole-slide images from the training set and evaluated on the same validation set used in the patch-level analysis. The patch ratios of each classifier were given as input to the whole-slide inference classifier. Low-risk images usually have a higher ratio of patches labeled as PUNLMP and low-grade patches. High-risk images typically have a higher ratio of high-grade and invasive patches. Fig. 2 shows the patch prediction level in four samples of whole-slide images (one sample per class). The *y-axis* represents the prediction ratio, the *x-axis* represents each WSI sample, and the bar colors are related to predicting classes. In other words, this figure illustrates how prediction aggregations are performed; for example, when a WSI contains the PUNLMP class, the ratio of predictions is expected to be higher for its class.

**Fig. 3:**
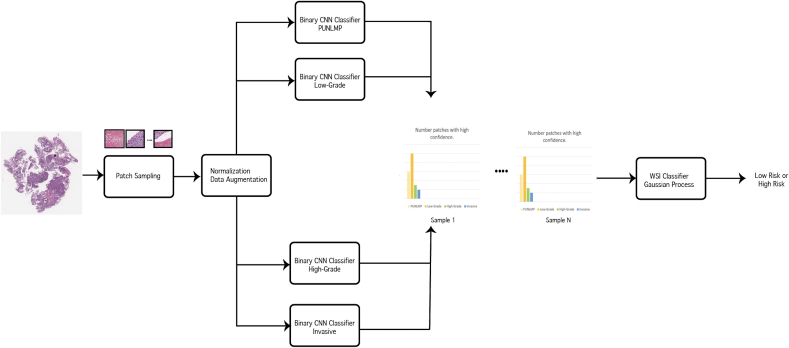
Patch prediction aggregation. Each patch label set should be in one of the four presented combinations. Other patch label combinations, where a patch can belong to more than one class, indicate a low confident patch and are eliminated. This particular case corresponds to low-grade risk.

Moreover, we integrated the output of four binary patch-level classifiers to keep high-confidence patches and exclude low-confidence and normal patches in the whole-slide inference step. This process in our proposed pipeline is shown in Fig. 3. If a patch is assigned to more than one of the labels, it indicates that the patch class label is unreliable and should be eliminated from the inference process. If all classifiers assign the label "others" to a patch, the patch is also eliminated. Our proposed inference method does not require hyperparameter tuning, as it does not rely on a threshold to eliminate low-confidence patches.

### Patient survival prediction

We analyzed the survival time of patients for low- and high-risk classes. Survival time was calculated from the date of diagnosis to the date of death for patients who did not survive or to the date when the Death Master File was queried for patients who survived.^21^ We generated Kaplan–Meier survival curves for patients predicted as high risk versus those predicted as low risk. A log-rank test was used to compare the survival between the two predicted groups, considering the follow-up time. We used the Cox proportional hazards model^25^ to estimate the effect size of our predicted risk group on patient survival.

### Evaluation metrics and statistical analysis

To measure the efficacy and generalizability of our approach, we evaluated our trained model on 182 independent whole-slide images (WSIs) from the NHBCS dataset and 378 WSIs from the TCGA dataset. We used precision, recall, and the F1-score as evaluation metrics. The confusion matrix was also generated for error analysis. In addition, 95% confidence intervals were computed using the bootstrapping method with 10 000 iterations for all the metrics.

## Results

### Classification of low- and high-risk groups

In Table 2, a summary is presented of our model's per-class and average evaluation metrics and the associated 95% CI for detecting low- and high-risk groups based on whole-slide images in the NHBCS test set. Our model achieved a weighted mean accuracy of 0.91, weighted mean precision of 0.91, weighted mean recall of 0.91, and weighted mean F1-score of 0.91 on the NHBCS test set.

**Table 2:** Model performance on 182 whole-slide images from the internal test set of NHBCS. The 95% confidence interval is also included for each measure.

Subtype	Accuracy	Precision	Recall	F1-score
Low-risk case	0.91 (0.86–0.94)	0.89 (0.83–0.94)	0.95 (0.91–0.98)	0.92 (0.88–0.95)
High-risk case	0.91 (0.86–0.95)	0.93 (0.88–0.98)	0.85 (0.78–0.92)	0.89 (0.84–0.94)
Average	0.91 (0.86–0.94)	0.91 (0.87–0.95)	0.91 (0.86–0.94)	0.91 (0.86–0.94)

**Fig. 4:**
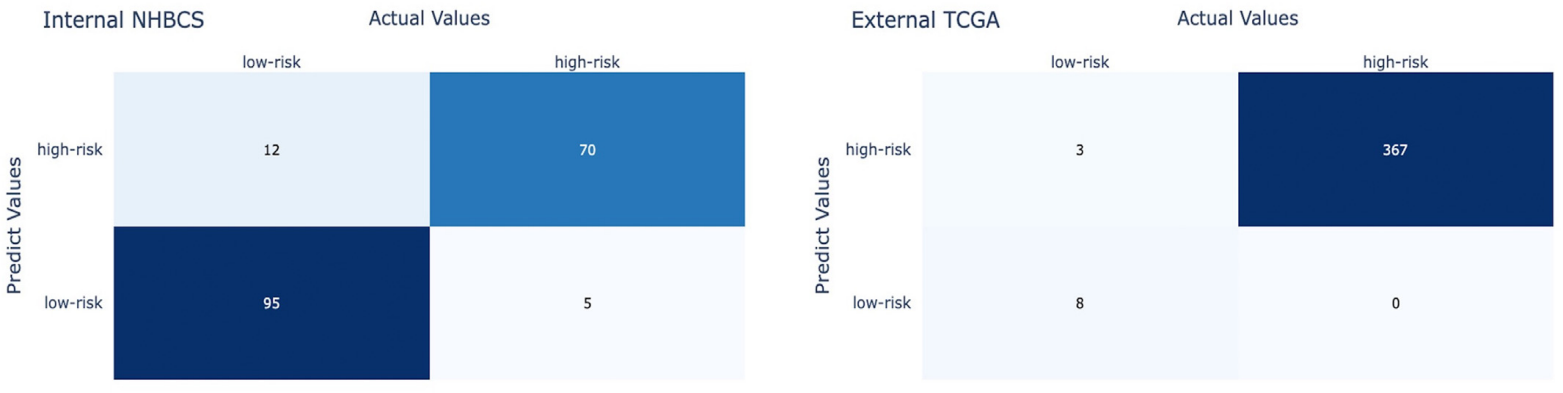
Each confusion matrix summarizes the model results compared to ground truth labels from pathologists on the (left) internal NHBCS test set and (right) external TCGA dataset.

Table 3 shows the performance summary of our model on whole-slide images from the TCGA database for the study of urothelial bladder carcinoma. Notably, each case in the TCGA dataset may have more than one whole-slide image. Therefore, the images for these patients were aggregated in our study. The cancer stage of all TCGA images was T2 and above, i.e., high risk, based on the patient metadata in the TCGA dataset. Although most patients in the TCGA cohort were in the high-risk group, high-risk histological patterns were absent on the histology slides of a few patients based on the evaluation of our pathologist expert. This is likely because only selected slides of each case were uploaded to the TCGA database, and the selected slides may not represent the entire tumor. Therefore, based on the tumor morphology of WSIs available for these cases, we considered these cases low risk. On the TCGA dataset, our model achieved a weighted mean accuracy of 0.99, weighted mean precision of 0.99, weighted mean recall of 0.99, and weighted mean F1-score of 0.99. The confusion matrices for our model on the NHSBC and TCGA test sets are shown in Fig. 4.

**Table 3:** Model performance on 378 whole-slide images from the external TCGA dataset. The 95% confidence interval is also included for each measure.

Subtype	Accuracy	Recall	Precision	F1-score
Low-risk case	0.99 (0.98–1.00)	1.0 (1.0–1.0)	0.73 (0.46–0.99)	0.84 (0.63–1.00)
High-risk case	0.99 (0.98–1.00)	0.99 (0.98–1.0)	1.0 (1.0–1.0)	0.99 (0.98–1.00)
Average	0.99 (0.98–1.00)	0.99 (0.98–1.0)	0.99 (0.98–1.00)	0.99 (0.97–1.00)

### Prediction of patient survival

**Fig. 5:**
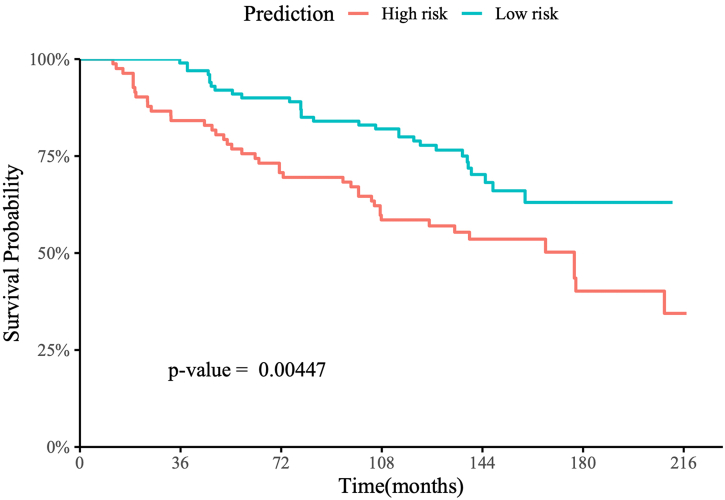
Kaplan–Meier survival curve of patients from the internal NHBCS test set with up to 216 months of follow-up.

**Fig. 6:**
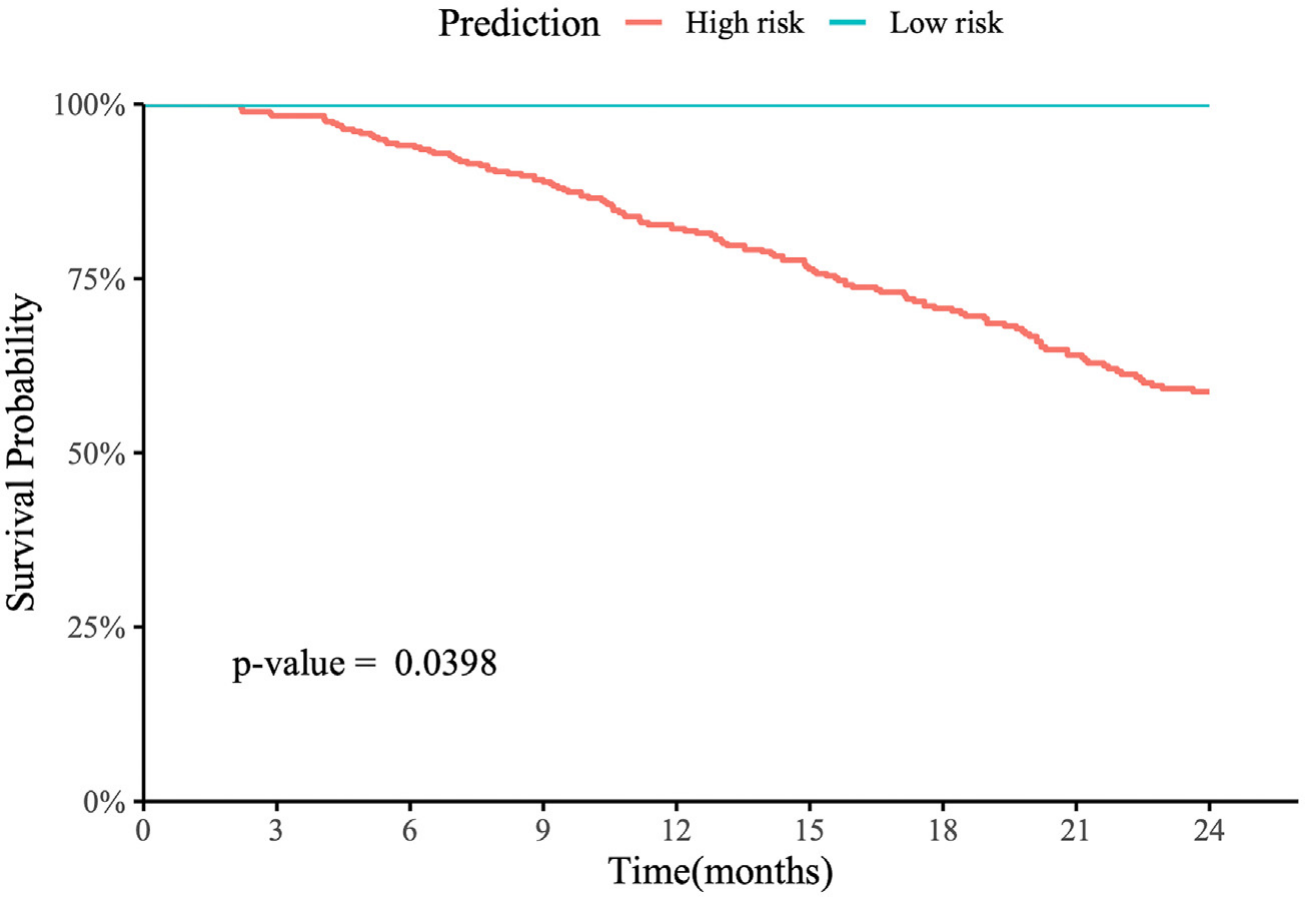
Kaplan–Meier survival curve of patients from the TCGA bladder cancer dataset with up to 48 months of follow-up.

Fig. 5 shows the Kaplan–Meier survival curve for patients from the internal NHBCS test set. The hazard ratio of overall survival using the risk group predicted by our model versus the tumor grade-defined risk groups for these patients is shown in Table 4. Fig. 6 shows the Kaplan–Meier survival curve of patients from the external TCGA test set.

**Table 4:** The hazard ratio of overall survival using the predicted risk group (predicted groups) versus the tumor grade-defined risk groups on patients from the internal NHBCS test set and the associated 95% CIs and p-values.

Predictor	Hazard ratio	p-Value
Predicted risk groups	1.958 (1.222, 3.137)	0.00523
Tumor grade-defined risk groups	1.945 (1.218, 3.107)	0.00537

For the internal NHBCS test set, patients predicted as high risk had worse overall survival than those predicted as low risk, with a log-rank p-value of 0.004 (Fig. 5). Additionally, NHBCS test patients were followed up for 216 months after their initial diagnosis, with a mean follow-up time of 123.8 months. The medium survival time of predicted high-risk patients was 177 months, while greater than 50% of the predicted low-risk patients survived until the end of their follow-ups. In the univariate Cox proportional hazards analysis using our predicted risk groups, the predicted high-risk group had an estimated hazard ratio of 1.958 (95% CI: 1.222–3.137, p-value=0.005) compared to the predicted low-risk group. This hazard ratio was slightly higher than the hazard ratio using the labels defined by the WHO/ISUP grading (Table 4).

Among 378 patients from the TCGA dataset, 367 were predicted to be high risk, and 11 were predicted to be low risk. Due to the small number of low-risk patients and the limited follow-up time, we limited our survival analysis to the first 24 months after the initial diagnosis. There was no death event reported during the follow-up for the low-risk group, and 35 death events were reported in the high-risk group in the first 24 months of follow-up, with a log-rank P-value of 0.04 (Fig. 6). Notably, due to the absence of events in the low-risk group, we could not estimate the hazard ratio and did not conduct Cox proportional hazards analysis on the TCGA dataset.

For the NHBCS test set, patients predicted as high risk had worse overall survival than those predicted as low risk, with a log-rank p-value of 0.004 (Fig. 5). The patients in the NHBCS dataset were followed up for 216 months after the initial diagnosis, with a mean follow-up time of 123.8 months. The median survival time of predicted high-risk patients was 177 months, while greater than 50% of the predicted low-risk patients survived until the end of their follow-ups.

## Discussion

The WHO has updated its bladder cancer grading guidelines several times since 1973 to align them more closely with disease recurrence and progression.^3^ Based on the most recent update from the WHO in 2016, PUNLMP, low- and high-grade stage T1 bladder cancers are categorized as NMIBC, and high-grade cases with stage T2 and above are classified as MIBC. The detection and classification of bladder cancer histologic patterns under the microscope is critical for accurate prognosis and the appropriate treatment of patients; however, this histopathological assessment is a time-consuming and challenging task and suffers from a high variability rate among pathologists. Therefore, patients with NMIBC can incorrectly be diagnosed as high-grade cases, which might result in unnecessary treatment or even surgery, which can affect patients' quality of life.^17^^,^^26^^,^^27^ In this study, we developed and evaluated a deep learning model to classify patients as high- or low-risk based on their whole-slide images to inform their prognosis and treatment. Our evaluation results on both internal and external datasets showed that this approach could potentially assist pathologists in their histopathological assessment, improve the accuracy and efficiency of a diagnosis, and ultimately improve patient health outcomes.

In recent years, deep learning models, such as convolutional neural networks or CNNs, have been applied to a variety of computer vision tasks as well as biomedical applications.^28^, ^29^, ^30^ CNN-based models have shown great promise in learning the morphological characteristics of different cancer types from histological images.^31^, ^32^, ^33^, ^34^, ^35^ A typical whole-slide image can range upwards of 150 000 by 150 000 pixels in size and occupy gigabytes of space. The large image sizes necessitate significant software engineering efforts in every histology image analysis pipeline stage, such as storage capacity, network bandwidth, computing power, and graphics processing unit (GPU) memory. Although analyzing slides directly at the WSI level can capture global structures/patterns and their relationships more accurately, this is not currently feasible on typical computational hardware and infrastructures. Therefore, our whole-slide histology image analysis framework analyzes high-resolution images at a patch level to tackle this feasibility problem. This pipeline relies on deep learning image analysis on small patches from the whole-slide images. The results are then aggregated through a confidence-based inference mechanism to classify the whole-slide images. While this approach is more limited in capturing large morphological structures and patterns across patches, it solves the computational challenges induced by the large size of histology whole-slide images and is manageable by common GPUs and computational hardware. Also, our approach is deterministic, and there is no stochastic step (e.g., random sampling) in the pipeline. As a result, our model generates identical results for a slide in different runs.

As part of this study, we investigated the development of a multiclass CNN model with four labels, including PUNLMP, low-grade, high-grade, and invasive cases. Although this model achieved a reasonable performance at the patch level (see Table S1 in the Supplemental Material), some classes, such as PUNLMP and invasive cases, achieved suboptimal results. This outcome indicates that a single multiclass model cannot effectively handle the complexities of this task and achieve good performance and generalization for all four classes. Notably, differentiating between PUNLMP and low-grade types has the lowest accuracy rate among clinicians due to their morphological similarities.^17^ In addition, high-grade bladder cancer cells are found in all stages of the disease. Therefore, in our study, we used the ResNet-18 architecture as a backbone for binary classifications to differentiate between various classes instead of a single multiclass model. Each of our four binary CNN-based patch-level classifiers focuses on one class and differentiates that class from other subtypes. Our patch-level classification results (Table S1) indicate the high performance of our approach for this patch-level classification, as all binary classifiers achieved an F1-score of more than 0.79.

As the primary whole-slide level classification outcomes, we focused on identifying low- and high-risk groups for bladder cancer based on histology slides, where the low-risk class includes PUNLMP, and low-grade cases and the high-risk class includes high-grade and invasive cases. The differentiation between these two risk groups has a significant clinical impact on patient prognosis and treatment. For whole-slide inferencing, we built a Gaussian process classifier based on the distribution of the classified patches from each slide.

To demonstrate the generalizability of our model,^36^ in addition to evaluating it on 182 whole-slide images in our internal test set from NHBCS. We also evaluated our approach on 378 whole-slide images from TCGA as an external test set. Our approach achieved a weighted average F1-score of 0.91 (95% CI: 0.86–0.94) on the internal NHBCS test set. Notably, the TCGA dataset is highly skewed towards high-risk cases (11 low-risk vs 367 high-risk cases). Our proposed model achieved the F-1 score of 0.84 (95% CI: 0.63–1.00) in low-risk cases and 0.99 (95% CI: 0.98–1.00) in high-risk cases. Considering the skewed distribution of the TCGA dataset, our model achieved a weighted average F1-score of 0.99 (95% CI: 0.97–1.00) on the entire TCGA dataset. TCGA metadata information showed that all the cases in this dataset belong to the high-risk class. However, a few cases were identified by our study's expert pathologists as low risk based on their histology images, likely because the selected slides included in the TCGA dataset may not represent the entire tumor.

The earlier study on 838 whole-slide images from NHBCS^21^ did not include any information on the types of specimens. Nevertheless, we do not believe the specimen types significantly affect our model. During annotation, the lesion areas with recognizable histology were included, and the areas with a crush, cautery, or processing artifacts were excluded. Furthermore, we did not include the tumor pathologic stage in this study. Therefore, the types of specimens do not significantly affect our model’s performance as long as the model recognizes the low- and high-grade features and invasion. The 378 whole-slide images from TCGA for external validation are all from cystectomy specimens.

Finally, we computed Kaplan–Meier survival curves for patients predicted as high risk versus those predicted as low risk for both the NHBCS and TCGA test sets. Patients predicted as high risk had worse overall survival than those predicted as low risk, with log-rank p-values of 0.004 and 0.039 on the NHBCS and TCGA test sets, respectively. Additionally, our predicted high-risk group in the NHBCS test had an estimated hazard ratio of 1.958 (95% CI: 1.222–3.137, p-value = 0.005) compared to the predicted low-risk group, which was slightly higher than the hazard ratio using the labels defined by the WHO/ISUP grading.

Of note, we did not sub-classify the high-grade lesions further based on the percentage of high-grade areas or the low- to the high-grade ratio in the current study. However, for future research, we plan to evaluate the performance of our model for borderline lesions (i.e., the majority of the lesion with low-grade morphology, while 5–10% of the area is occupied by high-grade foci). The nested variant of urothelial carcinoma was classified as invasive urothelial carcinoma in high-risk cases. Our model classifies invasive urothelial carcinoma based on not only the cytological features but also other histologic features, such as the growth pattern and the stromal reaction. In this study, we did not annotate invasive urothelial carcinoma with histologic subtypes due to the limited number of cases for these variants. In future work, we plan to evaluate the performance of our model for different histologic subtypes of invasive urothelial carcinoma. Finally, we plan to include additional relevant variants and factors in our pipeline, deploy our developed approach as part of a clinical decision-support system in clinical settings, and conduct a follow-up prospective clinical trial with appropriate clinical metrics to evaluate the clinical impact of this work on pathologist performance and patient health outcomes. We envision the deep learning model could provide a second opinion to the pathologists while reviewing the bladder cancer specimens, enhance the accuracy and efficiency of their performance, and improve patient outcomes.

## Conclusions

Our implementation achieved the weighted average F1-score of 0.91 (95% CI: 0.86–0.94) for the internal NHBCS test set and 0.99 (95% CI: 0.97–1.00) on the external TCGA test set, which implies that is possible to leverage Bladder cancer recognition using whole slide images. In future work, we plan to expand our model to distinguish between high-grade invasive and high-grade noninvasive classes, which is clinically helpful to determine the progression and reoccurrence of bladder cancer. Because we had a limited number of muscle-invasive cases in our datasets, building such a model for this differentiation was not feasible in the current study. We plan to collect additional data and develop new data augmentation techniques, such as generative adversarial networks (GANs), to tackle the dataset imbalance. Such techniques can mitigate the effects of unbalanced data by preventing overfitting and thus improving overall performance.^37^ In addition, we consider including vision transformers in our future pipeline to improve our high-resolution image encoding approach.^38^ In future work, we also want to deploy the developed model for histopathological characterization of whole-slide images for bladder cancer as a computer-aided diagnosis system in clinical settings. We also plan to conduct a follow-up prospective clinical trial to compare the model's performance to pathologists and evaluate its clinical impact on pathologists' performance. We envision the deep learning model could provide a second opinion to the pathologists while reviewing the bladder cancer specimens and enhance the accuracy and efficiency of their performance. Lastly, we have in mind to conduct a prospective study to validate our approach in clinical practice and evaluate its impact on health outcomes.

## Declaration of Competing Interest

The authors declare that they have no known competing financial interests or personal relationships that could have appeared to influence the work reported in this paper.

## Appendix A Supplementary data

Supplementary material Image 1
