# Supplementary material for: Bladder cancer prognosis using deep neural networks and histopathology images
Source: J Pathol Inform. 2022 Aug 28;13:100135. doi: 10.1016/j.jpi.2022.100135 (PMC9577122; doi:10.1016/j.jpi.2022.100135)
Supplement: Supplementary file 1 — Supplementary material [file mmc1.docx]

## **Supplemental Material**

**A1. Performance Measures**

The performance of our classification model is quantified by common evaluation metrics of Accuracy, Precision, Recall, and F1-score. The definitions of these metrics are as follows (TP: number of true positives, FP: number of false positives, TN: number of true negatives, FN: number of false negatives).

Accuracy = (TP + FN) / (TP + TN + FP + FN)

Precision = TP / (TP + FP)

Recall = TP / (TP + FN)

F1-Score = 2× (Precision × Recall) / (Precision + Recall)

### **Table S1: Patch-level Classification Results**

To address the challenge of classifying bladder cancer subtypes, we trained four CNN-based binary classifiers to differentiate each class at the patch level. We also developed a multi-class CNN model with these four labels using a ResNet-18 model. This multi-class patch-level classifier achieved the overall weighted F1-score of 0.80 (95% CI: 0.74-0.85). Also, a patch-level classifier trained on low- and high-risk patches achieved the weighted mean F1-score of 0.88 (95% CI: 0.83-0.92). The detailed results of these patch-level classifiers for each class are shown in the table below.

| **Patch-level classifier** | **F1-score** | **Precision** | **Recall** | **Accuracy** |
| --- | --- | --- | --- | --- |
| PUNLMP, low-grade, high-grade, IUC | 0.80 (0.74-0.85) | 0.80(0.74-0.86) | 0.80(0.74-0.85) | 0.80 (0.74-0.85) |
| PUNLMP vs others classifier | 0.94 (0.91-0.97) | 0.95(0.92-0.98) | 0.94(0.90-0.97) | 0.94(0.90-0.97) |
| Low-grade vs others classifier | 0.94 (0.91-0.97) | 0.85(0.79-0.90) | 0.94(0.90-0.97) | 0.86(0.80-0.90) |
| High-grade vs others classifier | 0.86(0.80-0.91) | 0.86(0.81-0.91) | 0.86(0.80-0.91) | 0.86(0.80-0.91) |
| IUC vs others classifier | 0.79 (0.64-0.92) | 0.97(0.94-0.99) | 0.96(0.93-0.98) | 0.96(0.93-0.98) |
| Low-risk vs high-risk classifier | 0.88 (0.83-0.92) | 0.88(0.83-0.92) | 0.88(0.83-0.92) | 0.88(0.83-0.92) |

**Table S2: Description of NHBCS dataset**

The Characteristics of 811 bladder cancer patients in the NHBCS dataset used in this study.

| **Variable** | **Type** | **Overall** | **Training** | **Testing** |
| --- | --- | --- | --- | --- |
| n |  | 811 | 525 | 286 |
| Age (SD) |  | 61.17 (10.33) | 59.82 (10.04) | 63.67 (10.41) |
| Sex (%) | Men | 586 (72.3) | 385 (73.3) | 201 (70.3) |
|  | Women | 225 (27.7) | 140 (26.7) | 85 (29.2) |
| Body mass index (SD) |  | 27.80 (5.03) | 28.17 (4.71) | 27.69 (5.12) |
| Family history of bladder cancer (%) | Yes | 40 (4.9) | 25 (4.8) | 15 (5.2) |
|  | No | 724 (89.3) | 453 (86.3) | 270 (94.8) |
|  | Unknown | 47 (5.8) | 47 (9.0) | 0 (0.0) |
| High-risk occupation (%) | Yes | 284 (35.0) | 128 (24.4) | 127 (44.6) |
|  | No | 258 (31.8) | 130 (24.8) | 156 (54.7) |
|  | Not available | 269 (33.2) | 267 (50.9) | 2 (0.7) |
| Smoke status (%) | Current | 246 (30.3) | 158 (30.1) | 88 (30.8) |
|  | Former | 397 (49.0) | 252 (48.0) | 145 (50.7) |
|  | Never | 160 (19.7) | 110 (21.0) | 50 (17.5) |
|  | Unknown | 8 (1.0) | 5 (1.0) | 3 (1.0) |
| Smoke pack-years (SD) |  | 40.01 (30.19) | 41.24 (31.06) | 37.88 (28.58) |
| UCC status (%)* | Confirmed UCC | 729 (89.9) | 456 (86.9) | 273 (95.5) |
|  | Not UCC | 17 (2.1) | 12 (2.3) | 5 (1.7) |
|  | Not available | 65 (8.0) | 57 (10.9) | 8 (2.8) |
| Muscle invasiveness (%) | Yes | 100 (12.3) | 58 (11.0) | 42 (14.7) |
|  | No | 705 (86.9) | 466 (88.8) | 239 (83.6) |
|  | Not available | 6 (0.7) | 1 (0.2) | 5 (1.7) |
| WHO 1973 classification (%) | Grade 1 | 336 (41.4) | 193 (36.8) | 143 (50.0) |
|  | Grade 2 | 166 (20.5) | 110 (21.0) | 56 (19.6) |
|  | Grade 3 | 161 (19.9) | 85 (16.2) | 76 (26.6) |
|  | Grade 4 | 65 (8.0) | 61 (11.6) | 4 (1.4) |
|  | Pathology material not reviewed | 17 (2.1) | 17 (3.2) | 0 (0.0) |
|  | Not available | 66 (8.1) | 59 (11.2) | 7 (2.4) |
| WHO/ISUP classification (%)* | CIS | 28 (3.5) | 15 (2.9) | 13 (4.5) |
|  | Papilloma | 2 (0.2) | 2 (0.4) | 0 (0.0) |
|  | PUNLMP | 201 (24.8) | 122 (23.2) | 79 (27.6) |
|  | LG-PUC | 225 (27.7) | 148 (28.2) | 77 (26.9) |
|  | HG-PUC | 196 (24.2) | 109 (20.8) | 87 (30.4) |
|  | Non-PUCHG | 53 (6.5) | 36 (6.9) | 17 (5.9) |
|  | Other | 42 (5.2) | 35 (6.7) | 7 (2.4) |
|  | Not available | 64 (7.9) | 58 (11.1) | 6 (2.1) |
| TNM Stage (%) | CIS | 41 (5.1) | 29 (5.5) | 12 (4.2) |
|  | 0 | 541 (66.7) | 347 (66.1) | 194 (67.8) |
|  | 1 | 123 (15.2) | 90 (17.1) | 33 (11.5) |
|  | 2 | 38 (4.7) | 23 (4.4) | 15 (5.2) |
|  | 3 | 22 (2.7) | 14 (2.7) | 8 (2.8) |
|  | 4 | 40 (4.9) | 21 (4.0) | 19 (6.6) |
|  | Not available | 6 (0.7) | 1 (0.2) | 5 (1.7) |
| P53 mutation (%) | Yes | 14 (1.7) | 14 (2.7) | 0 (0.0) |
|  | No | 186 (22.9) | 186 (35.4) | 0 (0.0) |
|  | Not available | 611 (75.3) | 325 (61.9) | 285 (100.0) |
| P53 intensity (%) | 3+ | 490 (60.4) | 298 (56.8) | 192 (67.1) |
|  | < 3 | 182 (22.4) | 120 (22.9) | 62 (21.7) |
|  | Not available | 139 (17.1) | 107 (20.4) | 32 (11.2) |
| P53 positivity (%) | 50% + | 323 (39.8) | 232 (44.2) | 91 (31.8) |
|  | < 50% | 322 (39.7) | 159 (30.3) | 163 (57.0) |
|  | Not available | 166 (20.5) | 134 (25.5) | 32 (11.2) |
| PTCH LOH positivity (%) | Yes | 119 (14.7) | 119 (22.7) | 0 (0.0) |
|  | No | 45 (5.5) | 45 (8.6) | 0 (0.0) |
|  | Not available | 647 (79.8) | 361 (68.8) | 285 (100.0) |
| First course of therapy (%) | No treatment | 39 (4.8) | 36 (6.9) | 3 (1.0) |
|  | TUR only | 577 (71.1) | 368 (70.1) | 209 (73.1) |
|  | Intravesical BCG* | 96 (11.8) | 63 (12.0) | 33 (11.5) |
|  | Intravesical Chemotherapy | 8 (1.0) | 5 (1.0) | 3 (1.0) |
|  | Radiation + Chemotherapy | 13 (1.6) | 6 (1.1) | 7 (2.4) |
|  | Cystectomy | 78 (9.6) | 47 (9.0) | 31 (10.9) |
| Mean survival time in months (SD) |  | 143.94 (55.94) | 159.89 (58.68) | 114.65 (35.11) |
| Death status at the 10-year mark(%) | Dead | 149 (18.4) | 95 (22.1) | 54 (18.9) |
|  | Alive | 662 (81.6) | 430 (77.9) | 232 (81.4) |

^*^UCC – urothelial carcinoma, HG-PUC –high grade papillary urothelial carcinoma, LG-PUC – low grade papillary urothelial carcinoma, PUNLMP – papillary urothelial neoplasm of low malignant potential, CIS – carcinoma *in situ*, BCG – Bacillus Calmette-Guerin
